# Afamin serum concentrations are associated with insulin resistance and metabolic syndrome in polycystic ovary syndrome

**DOI:** 10.1186/1477-7827-12-88

**Published:** 2014-09-10

**Authors:** Beata Seeber, Elisabeth Morandell, Fabian Lunger, Ludwig Wildt, Hans Dieplinger

**Affiliations:** Department of Gynecologic Endocrinology and Reproductive Medicine, Innsbruck Medical University, Anichstrasse 35, A-6020 Innsbruck, Austria; Department of Medical Genetics, Division of Genetic Epidemiology, Molecular and Clinical Pharmacology, Innsbruck Medical University, Schöpfstrasse 41, A-6020 Innsbruck, Austria; Vitateq Biotechnology GmbH, Innsbruck, Austria

**Keywords:** Afamin, Polycystic ovary syndrome, Insulin resistance, Metabolic syndrome

## Abstract

**Background:**

High plasma concentrations of the vitamin E-binding protein afamin have been previously shown to be associated with insulin resistance and metabolic syndrome. We set out to determine whether the concentration of afamin in the serum of women with polycystic ovarian syndrome (PCOS) is elevated in relation to the presence and severity of insulin resistance (IR).

**Methods:**

This cross-sectional study looked at 53 patients with PCOS and 49 non-PCOS patients. IR was diagnosed as a HOMA Index >2.4 and confirmed with a three-hour glucose tolerance test. Serum concentrations of afamin were determined using enzyme-linked immunosorbent assay (ELISA). Clinical characteristics, hormone and metabolic parameters were correlated to afamin concentrations.

**Results:**

Serum concentrations of afamin did not differ between women with PCOS and controls. When separated according to the presence of IR a significant difference in median afamin levels was seen between PCOS with IR and PCOS without IR (73.06+/-27.36 mg/L and 64.25+/-17.41 mg/L, p = 0.033). No difference in afamin levels was detected when comparing the few controls with IR and the controls without IR (76.20+/-27.96 mg/L and 60.44+/-21.03 mg/L, p = 0.235). On univariate analyses, afamin serum concentrations significantly correlated with BMI, triglycerides, HOMA Index, and AUC–Insulin. On multivariate linear regression analysis, only triglyceride concentration was seen to be an independent predictor of afamin. Subjects with metabolic syndrome had higher median afamin concentrations than did those without metabolic syndrome (77.43+/-28.60 mg/L and 65.08+/-18.03 mg/L, p = 0.010).

**Conclusions:**

Elevated afamin concentrations are associated with the presence of metabolic syndrome in young women and may potentially serve as an independent predictor for the development of metabolic syndrome in at-risk women, especially those with IR.

## Background

Polycystic ovary syndrome (PCOS) was first described in 1935 by Stein and Leventhal in patients with amenorrhoea, hirsutism, obesity and histological evidence of polycystic ovaries [1]. PCOS is a heterogeneous syndrome of unclear etiology with a prevalence of up to 20%, making it the most frequent endocrine disorder in women of reproductive age [2, 3]. There exist, however, no universal diagnostic criteria for PCOS [4], although most clinicians and researchers have accepted the definition published after the consensus conference in Rotterdam in 2003 [5]. Women are diagnosed with PCOS if they have at least two of the following criteria: a) oligo- or amenorrhoea, b) hyperandrogenemia or clinical signs of hyperandrogenism, c) sonographically diagnosed polycystic ovaries, *after* exclusion of other related disorders.

The pathogenesis of PCOS is a complex mixture of genetic and environmental origins [4]. Insulin resistance (IR) and compensatory hyperinsulinemia are observed in as many as 50%-60% of PCOS women [6]. Moreover, women with PCOS exhibit a four-fold higher incidence of metabolic syndrome. The prevalence of metabolic syndrome in PCOS is reported to be 8%-50%, depending on study design and population [7–9].

Metabolic syndrome has been increasingly diagnosed in the general population in recent decades. It is defined as a combination of risk factors for type 2 diabetes mellitus (T2DM) and cardiovascular disease (CVD), including dyslipidemia, hyperglycemia, abdominal obesity, and hypertension [10], three or more of which are required for the diagnosis [11]. According to data from the third National Health and Nutrition Examination Survey (NHANES III, 1988–1994), the overall prevalence of patients with manifest metabolic syndrome was 22%, increasing with age and depending on sex and ethnic origin [12]. If untreated, metabolic syndrome likely results in the early development of life-threatening sequelae such as T2DM, CVD and stroke [13–17]. It is not yet clear, however, to what degree these risk profiles are associated with cardiovascular outcomes in women with PCOS [18].

Very recently, the human plasma vitamin E-binding protein afamin was reported to be highly significantly associated with criteria for metabolic syndrome in three independent human general populations [19]. In that work, data from a prospective study design as well as corresponding data from afamin-transgenic mice suggest a role of afamin in the development of metabolic syndrome.

Afamin was discovered in 1994 as the fourth member of the human albumin gene family, which includes human serum albumin, alpha-fetoprotein and vitamin D-binding protein [20]. Afamin is a human plasma glycoprotein of 87 kD with 15% carbohydrate content and 55% amino acid sequence similarity to albumin. Circulating plasma afamin is primarily of hepatic origin [20]. Abundant concentrations of afamin have been described in plasma and other body fluids such as follicular, cerebrospinal and seminal fluid [21].

We previously demonstrated multiple vitamin E-binding sites of human afamin using a radio-ligand assay [22]. Afamin concentrations were found to correlate with those of vitamin E in follicular and cerebrospinal fluids, but not in plasma [21]. This may indicate a dissociation of afamin from its vitamin E carrier function in plasma, unlike its physiological carrier function in follicular and cerebrospinal fluids.

Prompted by these observations, we measured afamin concentrations in patients with and without PCOS and investigated the association between afamin, IR and metabolic syndrome as a first step in establishing afamin as a marker for the development of metabolic syndrome in PCOS women.

## Methods

### Subjects

Eligible subjects for this study were recruited from the Department of Gynecologic Endocrinology and Reproductive Medicine of Innsbruck Medical University. We approached reproductive-age women, 18–45 years of age, who were being evaluated at our clinic for irregular menses, hirsutism, and/or infertility between February and December 2009.

We classified the subjects based on the diagnosis of PCOS according to the Rotterdam Consensus criteria [5]. Biochemical hyperandrogenemia was defined as total testosterone concentration ≥0.4 ng/ml, as validated in our laboratory. We made further subgroupings based on the presence of IR defined as a HOMA Index of ≥2.4 [23]. In selected cases, the presence of IR was further confirmed by a modified three-hour glucose tolerance test with an AUCinsulin of >12,000 μIU*180 min/mL defined as IR. Metabolic syndrome was defined according to the NCEP-ATP-III guidelines as the presence of at least three of five criteria: fasting glucose ≥110 mg/dL, waist >88 cm, blood pressure ≥130/≥85, triglycerides ≥150 mg/dL, and HDL ≤50 mg/dL [24]. The control group consisted of women in whom PCOS and/or non-classical adrenal hyperplasia (NCAH) were excluded. Most of the patients in the control group were diagnosed with either male factor infertility or tubal infertility.

Exclusion criteria included a history of endometriosis, diabetes, thyroid dysfunction or known metabolic disorders, chronic inflammatory conditions, including rheumatologic conditions. None of the women included in this study were taking oral contraceptives, hormonal medications, or insulin-sensitizing drugs. The study was approved by the Ethics Committee of Innsbruck Medical University. All participating subjects provided written informed consent.

### Clinical and laboratory data collection

Clinical evaluation of the subjects included menstrual history and the presence of acne, hirsutism (evaluated with the Ferriman-Gallwey score) [25], seborrhoea, and androgenic alopecia. BMI, waist circumference, hip circumference, systolic and diastolic blood pressure as well as the appearance of the ovaries on ultrasound were recorded. Subjects underwent a peripheral blood draw after overnight fasting on cycle day 3–5 following a spontaneous or progesterone-induced bleed to determine a metabolic (total cholesterol, LDL cholesterol, HDL cholesterol, triglycerides, fasting glucose, fasting insulin) and hormone panel (luteinizing hormone (LH), follicle-stimulating hormone (FSH), estradiol (E2), dehydroepiandrosterone sulfate (DHEAS), testosterone, sex hormone-binding globulin (SHBG), free androgen index (FAI), prolactin and thyroid-stimulating hormone (TSH)). A separate blood sample collected for measurement of afamin was centrifuged at low speed, aliquoted and the serum stored at -80°C until analysis.

In addition, women with suspected PCOS underwent a 3 h, 75 g load oral glucose tolerance test (OGTT) for evaluation of the presence and degree of IR, as previously described [9]. In all women meeting criteria for PCOS late onset and heterozygous non-congenital adrenal hyperplasia were excluded with an ACTH stimulation test following overnight dexamethasone suppression, as previously described by Lejeune-Lenain [26].

### Laboratory analyses

Hormone concentrations were measured by specific commercially available immunoassays. Total testosterone was determined using a radioimmunoassay (RIA) method with a Coat-a-count In-vitro Diagnostic Test Kit (Diagnostic Products Corporation, Los Angeles, CA, USA) in a Siemens Immulite 2000 Immunoassay system. The sensitivity of this assay was 0.04 μg/L and the intra-assay and inter-assay coefficient of variation (CV) for the range of values pertinent to our patients was less than 10%. The free androgen index (FAI) was calculated as follows: total testosterone (nmol/L) × 100/SHBG (nmol/L).

Afamin serum concentrations were quantified in duplicate by custom-made sandwich-type ELISA using specific mono- and polyclonal antibodies recognizing human afamin (MicroCoat Biotechnologie, Bernried, Germany), as previously described [21, 22, 27]. Within-run and total CV were 3.3% and 6.2%, respectively, at a mean concentration of 73 mg/L [27].

The lipid profile including cholesterol and triglycerides as well as fasting glucose and insulin were determined using routine automated laboratory methods. Glucose was measured with the GOD-PAP method (glucose oxidase) and Roche/Hitachi 917 with an intra-assay CV of 0.9% and an inter-assay CV of 1.9%. Insulin was determined by electrochemiluminescence immunoassay (ECLIA) with an intra-assay CV of 1.9% and an inter-assay CV of 2.6%.

### Statistical analyses

Baseline characteristics of the two groups were compared using T test, chi-square, and Fisher’s exact test, where appropriate. Median concentrations of afamin were compared between groups using non-parametric tests, the Mann–Whitney test for two-group comparisons, and the Kruskal-Wallis test for multi-group comparisons. Bivariate relations between afamin and covariates were analyzed with Spearman’s Rank Correlation Coefficient, and linear regression was used to test which variables were independent predictors of afamin level. The pre-selected independent variables evaluated were BMI, fasting insulin, HOMA Index, triglycerides, and LDL cholesterol. Statistical analyses were performed with GraphPad Prism, Version 6.01 (GraphPad Software, San Diego, CA, USA), and SPSS 22 (SPSS, Inc., Chicago, IL, USA).

G*Power 3.1.2 (Kiel, Germany) was used to calculate the sample size needed for this study. Using our previously published normal population median of serum afamin of 64 mg/L [27] and setting an α error probability of 0.05 and power (1-β error probability) of 0.80, we performed a two-tailed sample size calculation to detect a difference of 12% between non-PCOS and PCOS subjects. A minimum of 90 subjects was needed to achieve adequate power to detect this difference.

## Results

We recruited 102 women for this study, 53 with PCOS and 49 without PCOS. Baseline characteristics, hormone concentrations and metabolic profiles of the study subjects are presented in Table 1. Women with PCOS were younger, had a higher BMI and waist circumference, and were more likely to have cycle irregularities, seborrhoea, and hirsutism. As expected, women with PCOS had higher concentrations of LH and testosterone, a higher FAI and LH/FSH ratio and lower SHBG concentrations than did non-PCOS women.

**Table 1:** **Anthropometric characteristics, hormone concentrations and metabolic profiles of subjects**

Parameter	PCOS (n = 53)	Controls (n = 49)	p
	mean ± SD or *median ± IQR.	mean ± SD or *median ± IQR.	
*Age yrs*	27.70 ± 5.33	32.14 ± 7.39	**0.001**
*BMI kg/m* ^*2*^	28.37 ± 7.14	23.278 ± 4.34	**0.001**
*Waist circumference cm*	99.33 ± 13.22	96.00 ± 9.90	**0.012**
*Blood pressure systolic mmHg*	124.39 ± 12.78	122.55 ± 12.76	0.532
*Blood pressure diastolic mmHg*	83.80 ± 12.22	79.74 ± 9.97	0.125
LH U/L*	8.00 ± 5.70	4.35 ± 2.98	**0.001**
FSH U/L*	5.85 ± 1.63	6.85 ± 3.25	**0.037**
LH/FSH*	1.51 ± 1.30	0.61 ± 0.37	**0.001**
E2 ng/L*	49.50 ± 22.25	39.00 ± 28.50	0.060
DHEAS mg/L*	1.94 ± 1.70	1.34 ± 0.95	**0.008**
Testosterone μg/L*	0.54 ± 0.36	0.22 ± 0.14	**0.001**
SHBG nmol/L*	35.45 ± 38.18	55.45 ± 35.65	**0.003**
FAI Index*	5.85 ± 6.14	1.78 ± 1.61	**0.001**
*Fasting glucose mg/dL*	87.30 ± 6.94	87.83 ± 8.68	0.734
Fasting insulin U/L*	9,8 ± 10,50	6,65 ± 5,92	**0.011**
*Total cholesterol mg/dL*	189.09 ± 33.10	181.43 ± 32.97	0.267
*HDL cholesterol mg/dL*	57.21 ± 16.79	60.87 ± 15.21	0.274
*LDL cholesterol mg/dL*	108.00 ± 27.5	96.50 ± 36.25	0.089
Triglycerides mg/dL*	101.00 ± 61.00	81.50 ± 44.75	**0.031**
AUC Insulin μIU*180 min/mL*	9935 ± 9762		
AUC Glucose mg/dl*180 min/mL	18606 ± 2762	16510 ± 1136	0.201
HOMA Index*	2.16 ± 2.37	1.39 ± 1.24	**0.013**
Afamin mg/L*	66.43 ± 19.60	62.33 ± 18.53	0.745

Values of variables are given as mean ± standard deviation (SD), or *median ± IQR. Statistically significant p values are shown as bold numbers. AUC: area under the curve.

Women with PCOS had significantly higher mean fasting insulin and triglyceride concentrations and a higher mean HOMA Index and were more likely to have IR (45.3% vs. 21.4%, p = 0.015), but not more likely to meet the criteria for MetS (20.75% vs. 8.11%, p = 0.103) than were those without PCOS.

**Figure 1:**
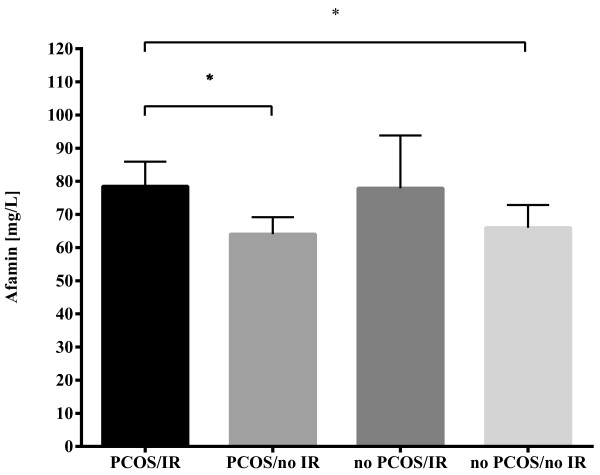
**Afamin concentrations in PCOS patients and non-PCOS controls classified according to insulin resistance.** Median serum afamin concentrations in PCOS patients and non-PCOS controls with or without IR. Subjects were subgrouped into PCOS patients with (n = 24) or without IR (n = 29) and into non-PCOS controls with (n = 9) or without IR (n = 33). Afamin concentrations were evaluated group-wise with the Kruskal-Wallis H test using Dunn’s correction for multiple comparisons. * indicates significance at the p < 0.05 level.

Median afamin concentration was 66.43+/-19.60 mg/L in the PCOS and 62.33+/-18.53 mg/L in the non-PCOS group, a statistically non-significant difference (p = 0.745, Table 1). Further separation of the subjects according to the presence of IR revealed a significant difference in afamin concentrations between the four groups (H(3) = 13.415, p = 0.004; Figure 1). Using Dunn’s multiple comparison test, we found significantly higher median afamin levels in PCOS with IR as compared to PCOS without IR and controls without IR, namely: PCOS with IR: 73.06+/-27.36 mg/L, PCOS without IR: 64.25+/-17.41 mg/L, controls without IR: 60.44+/-21.03 mg/L, p = 0.033 and p = 0.011. However, no difference in afamin levels was seen when comparing controls with IR and controls without IR (76.20+/-27.96 mg/L and 60.44+/-21.03 mg/L p = 0.235).

We further conducted a one-way analysis of covariance (ANCOVA) using afamin as the dependent variable, PCOS as the independent variable (two levels) and fasting insulin as the covariate. ANCOVA (between-subjects factor: PCOS, control) showed no main effects of PCOS *F*(1,92) = 0.115, p = 0.736), but revealed that it is the insulin concentration that mainly determines afamin value *F*(1.92) = 10.06, p = 0.002, ηp^2^ = 0.10. When controlling for fasting insulin, the adjusted means for afamin were 70.42 mg/L (SE = 2.77) and 69.14 mg/L (SE = 2.45) for the PCOS and the control group, respectively.

On individual correlations using the Spearman coefficient r, the following parameters correlated significantly with afamin concentrations: BMI (r = 0.231, p = 0.02), waist/hip ratio (r = 0.310, p = 0.019), fasting insulin (r = 0.313, p = 0.002), LDL cholesterol (r = 0.257, p = 0.013), HOMA Index (r = 0.310, p = 0.002), and triglycerides (r = 0.372, p = 0.001) as the only criterion of metabolic syndrome. No statistically significant correlation was seen between afamin and other parameters of metabolic syndrome, including waist circumference (r = 0.259, p = 0.052), systolic or diastolic blood pressure (r = 0.194, p = 0.085; r = 0.199, p = 0.077), fasting glucose (r = 0.055, p = 0.582) or HDL (r = -0.166, p = 0.111).

Afamin correlated with FAI (r = 387, p = 0.0001) but not with testosterone (r = 0.015, p = 0.879), SHBG (r = -0.134, p = 0.182), FSH (r = -0.156, p = 0.120) or E2 (r = -0.147, p = 0.145).

**Figure 2:**
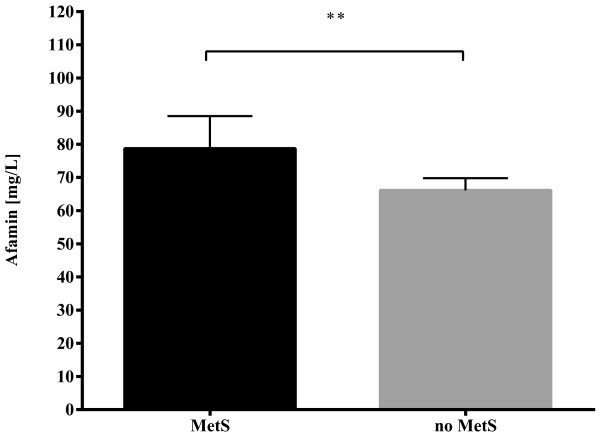
**Afamin concentrations and metabolic syndrome.** Serum afamin concentrations in all investigated subjects, independent of the presence of PCOS, but subgrouped into patients without (n = 76) or with (n = 14) metabolic syndrome. ** indicates significance at the p < 0.01 level.

However, on multivariable linear regression including as independent variables selected parameters that significantly correlated with afamin (BMI, LDL cholesterol, triglycerides, HOMA Index and fasting insulin) only triglyceride concentration was seen to be an independent predictor of afamin concentration (β coefficient = 0.271, p = 0.019, Durbin-Watson 1.468).Finally, we compared median afamin concentrations between those with and without diagnosed metabolic syndrome, independent of the presence of PCOS. We found markedly elevated afamin concentrations in women with metabolic syndrome versus without, values that were more than 12 mg/L higher than in women without metabolic syndrome, as shown in Figure 2 (77.43+/-28.60 mg/L and 65.08+/-18.03 mg/L, respectively, (p = 0.010)).

## Discussion

Women with PCOS have a higher prevalence of several risk factors for CVD, including dyslipidemia, IR, type 2 diabetes and hypertension, than do age-matched, non-PCOS women [28]. Although these risk factors are exacerbated by the concomitant obesity seen in some PCOS women, they are also present in lean, non-obese women with PCOS, many of whom exhibit IR [9, 29, 30]. Due to the elevated lifetime risk of developing metabolic syndrome and its subsequent atherosclerosis and CVD complications, the Androgen Excess and Polycystic Ovary Syndrome (AE-PCOS) Society has published specific recommendations for screening and management of women with PCOS [31]. Early identification of women at risk has clinical implications, because lifestyle modifications or medications such as insulin-sensitizing and/or cholesterol-lowering drugs may be initiated for primary CVD prevention.

The present study found no difference in serum concentrations of afamin, a vitamin E-binding protein, between women with PCOS and non-PCOS controls. When sub-grouping all investigated subjects according to the presence of IR, however, women with IR exhibited higher afamin concentrations, regardless of the presence of PCOS. A one-way analysis of covariance showed that it is fasting insulin and not the diagnosis of PCOS that correlates with afamin. Afamin was markedly higher in women already diagnosed with metabolic syndrome. These findings corroborate those of a previous population-based study that likewise showed that afamin concentrations were associated with the number of metabolic syndrome criteria in groups of men and women with mean age between 50 and 60 [19]. Despite evaluating a much younger cohort of women with an average age of around 30, the present study nonetheless found afamin levels to be similarly elevated. Age has, however, no or very little influence on afamin concentrations, as shown in a group of blood donors with different age subgroups [27]. Thus, our findings confirm that young women with PCOS are more likely to have metabolic syndrome, and afamin might serve as a discriminatory predictive parameter of IR in young PCOS patients. As such, PCOS can be considered a confounder in the relationship between afamin and IR.

This study confirms and extends a recently published independent investigation of afamin in PCOS patients [32]. The two studies differ, however, in study design and investigated key parameters. While Köninger et al. analysed retrospective data in a cross-sectional study, our patients were prospectively recruited. Furthermore, we were able to analyse the prevalence of IR based on HOMA Index as well as the prevalence of metabolic syndrome in both the PCOS- and the non-PCOS groups and provide information on the diagnosis of metabolic syndrome. In this way it was shown that, similar to the general population [19], afamin concentrations are associated with the prevalence of metabolic syndrome in PCOS patients. The present study did not find women with PCOS to have higher afamin levels than controls, as reported by Köninger et al. The differing results might be partially explained by the lack of prevalence data on IR and metabolic syndrome for the control group in the Köninger study, which could greatly influence the results if the control group consisted of a population almost exclusively having normal insulin sensitivity. Studies including larger numbers of subjects with documented IR levels and metabolic syndrome will therefore be necessary in future for final confirmation.

Of the laboratory parameters for metabolic syndrome evaluated, triglyceride level was the sole independent predictor of afamin concentrations. Interestingly, Dokras et al. reported that an elevated serum triglyceride/HDL cholesterol ratio was highly sensitive and specific for the detection of metabolic syndrome in women with PCOS [33]. The study by Kronenberg et al. [19] likewise found that afamin most strongly correlated with triglycerides and waist circumference in older populations of women and men.

Kronenberg et al. followed their subjects prospectively for five years and were able to show that each incremental increase in afamin of 10 mg/L increased the probability of being diagnosed with metabolic syndrome by 74%. Unfortunately, at this time we have no longitudinal data available for the women in the present cohort, but we are likewise following up the women in this study and re-evaluating afamin concentrations and components of metabolic syndrome in the hope that afamin may be useful as a predictive parameter for the development of metabolic syndrome in younger women with insulin resistance.

## Conclusions

It is only in recent years that more attention has been paid to the metabolic abnormalities of PCOS, and not just the syndrome’s implications for infertility and infertility treatment. As discussed in a recent review by Bates and Legro [34], understanding the true health implications of PCOS is difficult due to the heterogeneous nature of the condition and the lack of long-term cohort studies. Thus, large prospective longitudinal studies, ideally involving many ethnic groups, are needed to improve our understanding of the long-term health risks associated with PCOS. The results of the study at hand show that elevated afamin concentrations are associated with the presence of metabolic syndrome in young women and may potentially serve as an independent predictor for the development of metabolic syndrome in at-risk women, especially those with insulin resistance. For this purpose, however, prospective studies with sufficiently large cohorts of well-characterised PCOS women will have to be performed.
